# MHub.ai: A Standardized Platform for Reproducible AI Research in Medical Imaging

**DOI:** 10.21203/rs.3.rs-9480032/v1

**Published:** 2026-06-09

**Authors:** Leonard Nürnberg, Dennis Bontempi, Suraj Pai, Curtis Lisle, Steve Pieper, Ron Kikinis, Sil van de Leemput, Rahul Soni, Gowtham Murugesan, Cosmin Ciausu, Miriam Groeneveld, Felix J. Dorfner, Jue Jiang, Aneesh Rangnekar, Harini Veeraraghavan, Joeran S. Bosma, Keno Bressem, Raymond Mak, Andrey Fedorov, Hugo JWL Aerts

**Affiliations:** 1 Artificial Intelligence in Medicine (AIM) Program, Mass General Brigham, Harvard Medical School, Boston, MA, USA.; 2 Radiology and Nuclear Medicine, CARIM & GROW, Maastricht University, Maastricht, Netherlands.; 3 Department of Radiation Oncology, Dana-Farber Cancer Institute, Brigham and Women’s Hospital, Harvard Medical School, Boston, MA, USA.; 4 KnowledgeVis, LLC, Maitland, FL, USA.; 5 Isomics, Inc., Cambridge, MA, USA; 6 Department of Radiology, Brigham and Women’s Hospital, Harvard Medical School, Boston, MA, USA.; 7 Imaging Department, Radboud University Medical Center, Nijmegen, The Netherlands; 8 BAMF Health LLC, Grand Rapids, MI, USA; 9 Department of Radiology, Charité - Universitätsmedizin Berlin corporate member of Freie Universität Berlin and Humboldt Universität zu Berlin, Berlin, Germany; 10 Athinoula A. Martinos Center for Biomedical Imaging, Massachusetts General Hospital and Harvard Medical School, 149 Thirteenth St, Charlestown, MA, USA; 11 Department of Medical Physics, Memorial Sloan Kettering Cancer Center, New York, NY, USA; 12 Department of Diagnostic and Interventional Radiology, Technical University of Munich, School of Medicine and Health, Klinikum rechts der Isar, TUM University Hospital; 13 Department of Cardiovascular Radiology and Nuclear Medicine, Technical University of Munich, School of Medicine and Health, German Heart Center, TUM University Hospital

## Abstract

Artificial intelligence (AI) has the potential to transform medical imaging by automating image analysis and accelerating clinical research. However, research and clinical use are limited by the wide variety of AI implementations and architectures, inconsistent documentation, and reproducibility issues. Here, we introduce MHub.ai, an open-source, container-based platform that standardizes access to AI models with minimal configuration, promoting accessibility and reproducibility in medical imaging. MHub.ai packages models from peer-reviewed publications into standardized containers that support direct processing of DICOM and other formats, provide a unified application interface, and embed structured metadata. Each model is accompanied by publicly available reference data to confirm model operation. MHub.ai includes an initial set of segmentation, prediction, and feature extraction models for different modalities. The modular framework enables adaptation of any model and supports community contributions. We demonstrate the utility of the platform through comparative evaluation of lung segmentation models on public clinical data, and publicly release the generated segmentations and evaluation metrics as interactive dashboards to emphasize transparency and to enable case-level inspection. By enabling side-by-side benchmarking with identical execution commands and standardized outputs, MHub.ai reduces technical friction for model execution, evaluation, and comparison.

## INTRODUCTION

Artificial Intelligence (AI) methods, including convolutional neural networks, have demonstrated capabilities in interpreting medical imaging in radiology, pathology, and other domains ^1–6^. Multiple studies have shown that these models can perform tasks such as segmentation, classification, and feature extraction with an accuracy comparable to human experts ^7,8^. Despite this progress, integration into real-world clinical and scientific workflows remains limited. Barriers include inconsistent implementations, complex installation procedures, stringent preprocessing requirements, unclear configuration parameters, arbitrary output formats, and incomplete documentation ^9–11^. Additionally, although DICOM is the standard format in clinical environments, many AI models are built on alternative image formats, which requires manual conversion and organization. Together, these factors make it harder for other investigators to use these models, hinder model comparability, and undermine reproducibility. Consequently, most published AI models are not used by third-party researchers and are rarely implemented in clinical practice ^12,13^.

**Figure 1:**
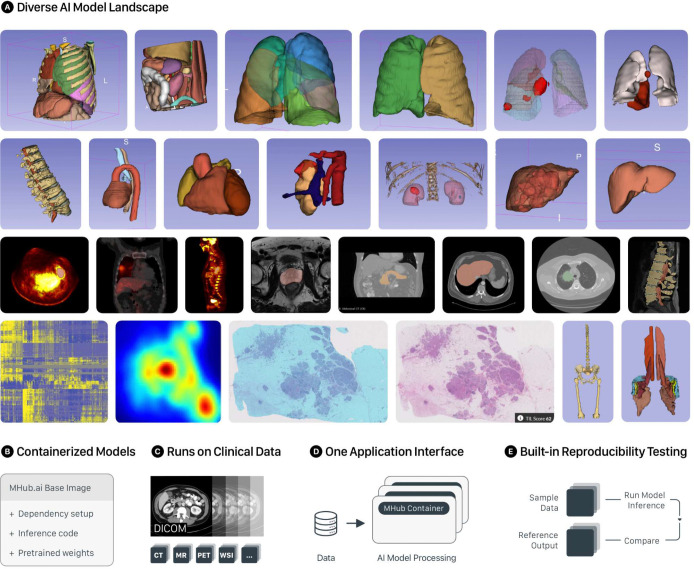
MHub.ai overview. *a) Diverse model landscape*. Representative examples of the models included in the MHub.ai platform for various imaging modalities, domains, and tasks. Segmentation, prediction, and feature extraction models are supported by the framework and are represented in the model repository More than half of the models overlap with others in one or more outputs, increasing the available choices. *b) Containerized.* All models are available as self-contained images, run in a shielded container sandbox, require no installation, and can be easily removed. *c) Run on clinical data.* The framework supports several input modalities, such as Computed Tomography (CT), Magnetic Resonance Imaging (MRI), Positron Emission Tomography (PET), Whole Slide Imaging (WSI), X-ray, and image formats such as Joint Photographic Experts Group (JPG) or Portable Network Graphics (PNG), and can be extended to support additional modalities and file formats. *d) One application interface.* The MHub.ai framework adapts existing AI pipelines to the clinical imaging standard format DICOM, enabling direct integration with existing public and private data providers as well as third-party tools for visualization, inspection, organization, and analysis. *e) Built-in reproducibility testing*. A task-specific real-world example input and reference output are available for all models. The test engine included in each model container can run the model on the sample input and compare the output with the reference.

MHub.ai was developed to reduce these barriers at inference time. The platform provides containerized AI models with a standardized application interface, built-in DICOM compatibility, and reproducibility tests (Figure 1b,c,d,e). The model repository contains various public models for multiple input modalities, imaging domains, and tasks (see Figure 1a,c), and the underlying framework provides tools to unify the application interface across models and to integrate with existing data providers, data organization, and visualization tools using established standards (Figure 1d). By aligning the packaging, distribution, setup, and execution of AI models and providing automated reproducibility tests, MHub.ai reduces technical friction for model execution and enables third-party investigators to use, evaluate, and compare models consistently, supporting systematic benchmarking and lowering barriers to clinical adoption.

In this work, we first describe the platform architecture and framework, provide an overview of the diverse model repository, and demonstrate the utility of standardized public models by comparing three lung segmentation models on public clinical imaging data.

## RESULTS

### Platform Architecture

**Figure 2:**
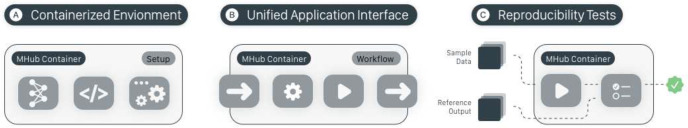
MHub.ai platform principles. *a) Containerized models.* Each AI model is packaged in an isolated container that includes inference code, pre-trained weights, dependencies, libraries, and the runtime environment for platform-independent execution with no individual setup required. *b) Unified application interface.* A standardized interface provides native DICOM handling, workflow orchestration, and consistent input and output structures across models. *c) Reproducibility tests.* Public sample data and reference outputs are provided for every model. The framework’s test engine runs each model on specific sample data, compares the outputs with the reference data, and reports the test results in a human- and machine-readable format.

MHub.ai is implemented as a modular platform based on three primary components as illustrated in Figure 2: a containerized environment to bootstrap the setup of the model, a unified application interface and structured metadata to streamline interaction with the model, and reproducibility tests to validate the pipeline.

#### Containerized environment.

Each model is packaged on top of the MHub.ai base image and bundles everything required to run the model: the inference source code, model weights, dependencies, and runtime environment (Figure 2a). Containerization enables portability, cross-platform compatibility and isolation. All models share the same base image, reducing the disk footprint through layer sharing ^14^.

#### Unified application interface and structured metadata.

All containers use a unified application interface, which streamlines interaction with the models by standardizing inputs and outputs (IO), configuration, and execution. All containers accept clinical images in native DICOM format and provide outputs using standardized representations for each output type, for example, DICOMSEG ^15,16^ for segmentations and JSON/CSV for tabular data, referencing the unique identifier of the image. The framework manages data flow using semantic annotations and configurable workflows (Figure 2b). Each model is accompanied by structured metadata that documents model architecture, intended use, training and test datasets, performance evaluation and limitations, descriptors for input and output data, supported input modalities, contrast-related training information, recommended maximum slice thickness, licensing information, references to source code and publications, and example images.

#### Reproducibility tests.

Each model includes public, real-world example inputs and reference output data stored in Zenodo ^17^ (Figure 2c). The test engine included in every model image loads the data into the container, runs the model on the sample input data, compares the generated output with the reference output, and produces machine- and human-readable reports, including contextual differences such as missing or extra files, Dice score comparisons for segmentation outputs, and key-value comparisons for structured outputs. All models are tested during the contribution process and before publication in the model repository.

### Model Repository

**Figure 3:**
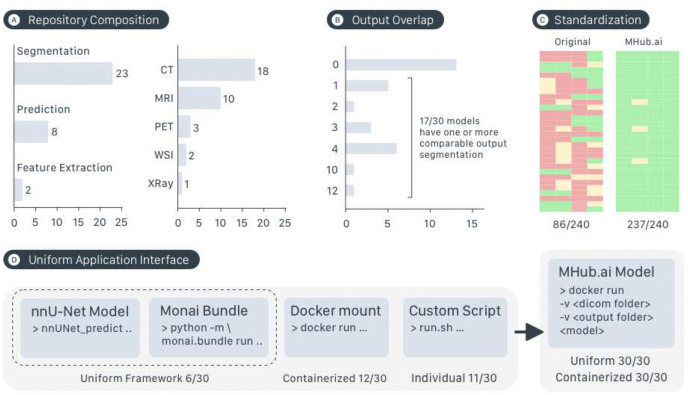
Diverse and uniform model landscape. *a) Repository composition.* Distribution of integrated models by task type and supported imaging modalities, illustrating the diversity of models integrated into the platform (n = 30). *b) Output overlap.* The number of models that share a set number of segmentation output. For 17 models share at least one segmentation label with another model, enabling direct cross-model comparison without additional data transformation. *c) Standardization*. Comparing standardization for input format, output format, inference application interface and user documentation between the original model publication and the MHub.ai distribution of the same model. Green = standardized / accessible (2 points), yellow = partially standardized / accessible (1 point), red = not standardized / accessible (0 points). *d) Uniform application interface*. Heterogeneous invocation mechanisms across original model distributions, including framework-specific commands, container-based execution, and custom scripts, are harmonized into consistent containerization and unified model execution.

MHub.ai currently integrates 30 peer-reviewed, open-source AI models for medical imaging, covering segmentation (n = 22), prediction (n = 8), and feature extraction (n = 2) tasks across CT (n = 18), MRI (n = 10), PET (n = 3), X-ray (n = 1), and pathology (n = 2) modalities (Figure 3a).

Standardized output representations enable cross-model comparability. Among these models, 17 (56.7%) share at least one segmentation output with another model (Figure 3b), enabling direct pairwise comparison without additional data transformation or relabeling.

In their *original distribution formats*, models show low standardization (36%) across input and output formats, application interfaces, and user guidance (Figure 3c). Twenty of the 30 models support only non-DICOM input formats, requiring manual conversion from clinical DICOM data. Only 7 of the 20 segmentation models provide DICOM-compatible DICOMSEG outputs with semantic annotations. Application interfaces for running model inference vary across framework-specific commands (for example, nnU-Net or MONAI pipelines), custom scripts, and container-specific commands. Only 13 models provide a user guide explaining how to run inference, 8 models provide some documentation, and 9 models provide no documentation at all.

In contrast, *after integration into the MHub.ai format*, all models operate directly on standard DICOM input by default and generate uniform DICOMSEG files for all segmentation tasks and JSON files referencing the DICOM image. All models now share a uniform application interface (Figure 3d) for inference and reproducibility testing. Model documentation, user guidance for running inference, task-specific example data, and reference outputs for automated reproducibility testing are available for all models. Overall, integration into the MHub.ai format improved standardization across input data format, output format, model inference, and user documentation from 86 of 240 available points by 275% to 237 points.

### Integration with external tools

The standardized model interface and DICOM-native IO design enable integration with established external imaging platforms, research tools, and visualization tools. We developed the community extension MHubRunner for 3D Slicer ^18^, which retrieves available MHub.ai models via a public web-API and enables model selection, execution, and visualization within the 3D Slicer environment. Because MHub.ai models share a uniform interface and standardized output formats, no model-specific adaptation is required for integration. Additionally, the standardized segmentation outputs and reference to the original image enable direct use within web-based viewers such as OHIF ^19^, supporting flexible visualization and integration in existing imaging environments, as we will demonstrate in the next section.

### Infrastructure-enabled comparative evaluation: lung segmentation use case

**Figure 4:**
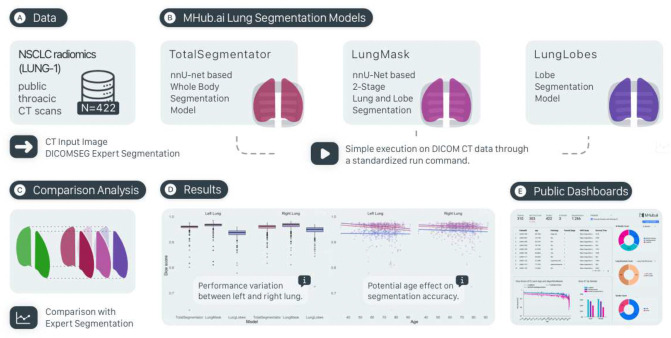
Streamlined comparison of lung segmentation models. a) *Public dataset input*: A total of 422 chest CT scans from the publicly available NSCLC-Radiomics dataset ^20^ were processed, with expert segmentation available for 303 scans. b) *Unified Model Execution*: The three segmentation models were executed directly on the downloaded DICOM files using a standardized Docker run command, while the MHub.ai framework automatically performed DICOM preprocessing and model execution. c) *Comparative analysis*: The standardized segmentation results of the three models were compared with expert annotations using Dice similarity, for the right and left lung, to evaluate and compare the performance of the different models. d) *Insights into model differences*: Agreement between automated model segmentations and expert reference annotations is measured using the Dice score metric and shown by model for the left and right lungs (left), and plotted against age for each lung across models (right). In both views, Dice scores are high, with a stable relative ordering of models. e) *Public dashboards:* Results are made publicly available as interactive dashboards to recreate and extend our analysis results and perform visual case-level inspection entirely in the web browser.

To demonstrate how standardized packaging and interfaces enable reproducible model comparison, we evaluated three independently developed lung segmentation models, integrated into MHub.ai, using expert annotations on a shared public clinical dataset (Figure 4a).

The standardized execution interface provides a uniform inference command. The directory containing all DICOM files, with no prior processing, is specified as input. The output is organized in a shared output directory. The following commands were used to generate the segmentations:


export input = /path/to/input/ct/scan/dicom/files/
export output = /path/to/output/directory/

docker run –rm -it -v $input:/app/data/input_data:ro -v $output:/app/data/output_data
mhubai/**totalsegmentator**:latest

docker run –rm -it -v $input:/app/data/input_data:ro -v $output:/app/data/output_data
mhubai/**lungmask**:latest

docker run –rm -it -v $input:/app/data/input_data:ro -v $output:/app/data/output_data
mhubai/**gc_lunglobes**:latest


Internally, DICOM separation into CT images, preprocessing, workflow orchestration, postprocessing, and output organization are handled automatically by the MHub.ai framework. No manual data preparation or model configuration is required (Figure 4b). The standardized output representations enabled harmonization of model predictions to a common semantic format and direct quantitative comparison (Figure 4c). All three models achieved high Dice similarity relative to expert segmentations. Small but statistically significant differences were observed in selected stratifications. This use case demonstrates how harmonized inference interfaces and standardized outputs enable fast and reproducible, side-by-side evaluation of independently developed AI models on clinical DICOM data (Figure 4d). Setup-free case-level inspection of the public dataset, the segmentations generated by the models and the Dice scores used throughout our evaluation is available through a public interactive dashboard (Figure 4e).

## DISCUSSION

MHub.ai defines a standardized format for inference-time distribution of medical imaging AI models. Although many models are publicly available, third-party adoption and reuse remain limited due to heterogeneous implementations, complex environment setup, limited guidance and documentation, inconsistent data handling, and uncertain reproducibility. By combining containerization, a unified application interface, native support for clinical imaging data formats, structured metadata, and built-in reproducibility testing, MHub.ai provides a consistent distribution and execution format for independently developed models.

For AI models to be reused by third parties, they must be easy to discover, set up, and execute. For model training, this has been addressed through widely adopted frameworks such as TensorFlow ^21^, PyTorch ^22^, nnU-Net ^23^, and MONAI ^24^, which provide standardized abstractions and interfaces. However, to the best of our knowledge, an equivalent system-level approach for inference-time distribution does not exist. Existing model repositories, such as the MONAI Model Zoo ^25^, Hugging Face ^26^ and BioImage Model Zoo ^27^, facilitate model discovery but do not enforce standardized execution interfaces, uniform data handling, or complete structured metadata and documentation. Individual models such as TotalSegmentator ^28^ demonstrate that straightforward installation and consistent inference interfaces for multiple segmentations are possible and have resulted in wider adoption by third-party researchers. However, such examples remain rare. MHub.ai focuses exclusively on inference-time standardization. Models operate independently of training ecosystems and can coexist with existing distribution methods while enforcing consistent execution and validation standards. This design offers flexibility for model developers while ensuring a consistent user experience running such models. To demonstrate this capability, we implemented 30 independently developed models across various tasks, image modalities, model architectures, frameworks, file formats, and dependencies in the MHub.ai format, including a model from the MONAI Model Zoo ^25^ to illustrate compatibility with existing repositories. The format proved flexible enough to containerize all models while improving standardization of input, output, interface, and documentation from 36% to 99%.

Reliable integration of AI models into research and clinical workflows requires evaluation under conditions relevant to the intended use case. Reported model performance metrics often vary across datasets and evaluation protocols, making direct comparison difficult. It has therefore been suggested that AI models should be evaluated within the target population rather than relying solely on benchmark metrics reported on heterogeneous datasets ^29^. Evaluating models for a common task on the same dataset enables more meaningful comparison under identical conditions. Our platform facilitates this in two ways: through containerization and by harmonizing the application interface we reduce the barrier to run a model over a dataset; by emphasizing overlap between models and by providing consistent and directly comparable output for same tasks. As an illustrative example, we selected lung segmentation on CT scans, where accurate delineation is critical for diagnosis, quantitative imaging, and radiation treatment planning, as it ensures effective dosing while sparing healthy tissue ^30,31^. We start with a public dataset of clinical CT images and generate lung segmentations using three different AI models. Although this may seem straightforward, it typically requires understanding how each model works, installing and configuring each model separately, converting data from DICOM to NIFTI for TotalSegmentator, and writing custom automation to run each model on each input image using their individual CLI arguments. However, the uniform interface of MHub.ai consolidates all these steps into a single command executed once per model, reducing setup and preparation overhead to nearly zero. By simplifying the process, we not only save time but also lower the barriers to quickly evaluate and compare independently developed AI models on any clinical data.

A uniform landscape of models makes them easy to use. However, to allow the underlying framework to improve over time and to keep all models synchronized, model containers cannot remain static but must be updated periodically while maintaining reproducible functionality. For example, replacing the internal DICOM conversion tool with a faster variant in the future might work well for most models but could cause some models to fail or, even worse, generate different output. To prevent this, we developed a testing mechanism embedded directly in the framework. Every model contains a pair consisting of a public, task-specific, real-world sample image and the expected reference output. Whenever the framework is updated, the test engine loads the sample data, generates a report of all findings, and prevents the model from being pushed to the public repository if discrepancies are found. The example input and output further enhance usability by illustrating the expected input format and providing a precise example output, enabling validation of model integration into pipelines before execution.

The proposed approach and design decisions have introduced some limitations to the platform in its current state that should be considered. The current implementation relies on Docker containers, which may require administrative privileges and may not be permitted in all computing environments. However, the framework itself is independent of the container runtime and could be adapted to alternatives such as Singularity ^32^. Compliance with metadata requirements and reproducibility tests introduces additional effort for model contributors, although reusable modules and detailed documentation are provided to simplify integration. In addition, MHub.ai operates independently of and alongside other existing packaging approaches, reducing vendor lock-in. Current format support does not yet cover all imaging modalities (e.g., whole slide pathology segmentations beyond TIFF). Reproducibility tests are performed on a single sample and cannot guarantee coverage of every edge case. Many models also require GPU acceleration, which may limit deployment on some systems.

MHub.ai is designed as an extensible platform. Future developments may include support for additional container runtimes, expanded modality support, and automated performance evaluation modules to enable standardized benchmarking. Community-driven contributions through open-source development will further increase the range of supported models and reusable modules. By lowering technical barriers to model execution and comparison, MHub.ai provides infrastructure for more reproducible and scalable evaluation of AI methods in medical imaging.

## METHODS

**Figure 5:**
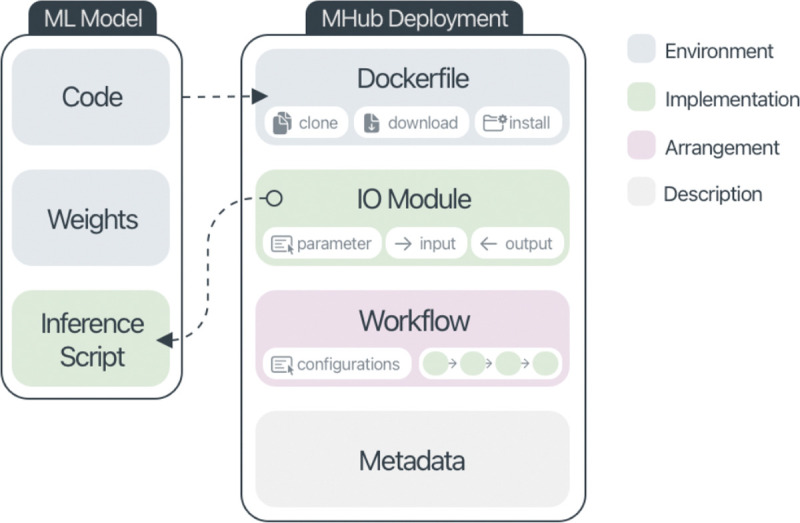
The MHub.ai Model Deployment Format. The MHub.ai deployment template is organized into environment, implementation, arrangement and description. System dependencies, model inference code and the trained weights are bundled in a container defined by a Dockerfile. The inference script is integrated through an IO Module adapter, including annotations for the required input data, a description of the output data and configurable parameters. The DICOM to DICOM execution is arranged in a workflow file, where configurable parameters and the sequential execution of IO Modules are defined. Model metadata is provided separately and in a defined schema.

We describe our AI model bundling strategy (see Figure 5), which includes the MHub.ai framework, metadata, reproducibility tests, the CI/CD pipeline supporting platform contributions, and the dataset and model evaluation for the use case. Additional details on the motivation for the design choices and the technical implementation are provided in the appendix of the technical preprint ^33^.

### Model Containerization

All models are containerized using Docker. Pre-built, ready-to-use container images are publicly available for all models on Docker Hub (repository: mhubai/<model_name>). We provide a base image with common tools and our framework pre-installed and configured. The base image depends on the availability of CUDA GPU drivers on the host system that match the available hardware and are deliberately not included. We have defined a Dockerfile structure that includes the following steps: start from `mhubai/base:latest`, install the model dependencies, install the model source code, download the model weights, include the MHub.ai wrapper, and set the MHub.ai default entry point (see **Environment**, Figure 5) and is enforced as part of our continuous integration (CI) pipeline.

### MHub.ai Framework

The MHub.ai runtime environment is implemented as a Python package. Modularization and configurability are core concepts. Each task is implemented as an IO Module (see **IO Module**, Figure 5) and corresponds to an IO Module Python class. The framework provides Python decorators to conveniently annotate configuration parameters, input, and output data, which are automatically resolved into absolute file-paths at runtime.

Data exchange between IO Modules is managed by the data management layer of the framework. For each IO Module, required inputs and generated outputs are declared using semantic queries and descriptors. A semantic descriptor registers a module’s output with the runtime engine and includes a file type declaration followed by descriptive key-value pairs. A semantic query uses the same syntax but can include operators and placeholders. Within an IO Module, variables are provided for each input, output, and configurable parameter through decorators. The engine resolves these at runtime into absolute file paths and tracks related metadata in a local graph. It searches the graph for files matching the specified type and metadata, supplies the resolved files as module inputs, and generates absolute file paths for declared outputs. This mechanism creates a consistent directory structure within the container. Semantic declarations keep IO Modules universal and independent of custom naming conventions and directory layouts. The use of readable descriptors makes data flow easier to inspect and understand, and all intermediate files remain accessible and can be included in the model output.

The framework includes 18 base modules to support the processing of medical images: importers that search the input directory and import relevant files into a uniform semantic structure; filters that selectively refine datasets based on instance attributes or file availability; converters that convert images between formats such as NIFTI, MHA, TIFF, PNG, DICOMSEG, and RTStruct; exporters that compile and output metadata and custom reports; and organizers that orchestrate the selection and format of output. Tools needed for these processes, e.g., dicomsort ^34^, plastimatch ^35^, dcm2niix ^36^, and dcmqi ^16^, are pre-installed in the base image for all models. The AI inference code is called from a custom IO Module (see **Implementation**, Figure 5) that wraps the original AI model and defines the configurable parameters and input and output data. To annotate the output of segmentation models, the framework includes a keyword-based lookup table with consistent DICOMSEG annotations. We assigned a unique identifier, e.g. HEART or LEFT_UPPER_LUNG_LOBE, to each ROI, mapping them to a human-readable name, RGB color, and semantic annotation codes that include category, type, and optional modifiers. For anatomical findings such as tumors, cysts, or calcified plaques, an intuitive annotation format, e.g. LEFT_LUNG+TUMOR, is provided to encode the surrounding tissue.

IO Modules are organized into workflows executed by the runtime engine. A workflow includes a label, description, sequential order of IO Modules, and configuration parameters in a single YAML file (see **Workflow**, Figure 5). For each MHub.ai model, the required default workflow operates on DICOM inputs and generates DICOM output where applicable (e.g., for all segmentation models). Additional workflows (e.g. to support alternative file formats and structures) can be included within the model or provided by the user.

All MHub.ai models use the same application interface. A docker run command creates a container from an MHub.ai model image and mounts the input and output directories. Because the input and output mount points inside the container are standardized across MHub.ai models, the run command differs only by the model name. Inside the container, the run engine (mhub.run) provided by the MHub.ai framework is executed. The run engine offers a CLI interface to specify the workflow (--workflow default), run utilities (--utility dicomsort), overwrite configurable parameters (--config:module.parameter=value), enable output logging (--print), and enable debugging (--debug, --stop-on-error). All CLI parameters are optional. If no workflow is specified, the default workflow is executed.

Workflows are not limited to the included core IO Modules. Custom modules use the same interface and decorator conventions as standard modules. Custom modules can be directly included in the model definition. This is how the model’s inference is called by implementing a wrapper IO Module that acts as an adapter and enables the MHub.ai run engine to call the inference as part of a workflow. Such a custom module is available only to the specific model it is shipped with. For reusable IO Modules, the framework provides an extension API to include IO Modules from third-party repositories at build time. When a workflow is loaded, the framework automatically discovers and registers all available modules (core modules, model-specific modules, and modules supplied through installed extension bundles) so they can be referenced by name in the workflow configuration file. Extension bundles must follow the required directory and module structure; bundles from unlisted repositories are supported but trigger a warning. An official list of supported extension bundles is available in the public model repository. This mechanism enables workflows to incorporate domain-specific or model-specific processing steps, supports reuse of common components, and allows functionality to be extended without modifying the underlying framework.

### Metadata Schema

The model's metadata is provided in a single meta.json file (see **Metadata**, Figure 5). The structure, required and optional keys, and value types are defined in a JSON schema ^37^ that is publicly available in our repository and is automatically verified as part of our CI pipeline.

### Reproducibility Testing

Each model contains a sample input for each workflow, a representative input chosen specifically for the model. Wherever possible, we use real sample images from a public dataset. The model is run on the sample image to produce a reference output, which is stored on Zenodo ^17^ along with the sample input. As part of the MHub.ai framework, we provide a comparison tool with each model container that loads the sample and reference data, runs the model and generates an automatic report. Multiple comparison checks are performed: at directory level, where missing and additional files are listed, at file level, where differences in file size are recorded, and at content level depending on the file type. For segmentations, a Dice score below 0.99 compared to the reference segmentation is reported as a deviation. For structure files such as JSON or CSV, missing and additional key and value differences are reported. The report is created as a machine and human readable yaml file. As part of the contribution process, an independent reproducibility check is performed for each model. The results are publicly available on Github. The reproducibility tests are not limited to the sample included with each model. Any public or private set of input and output data can be loaded into the test pipeline, allowing third-party users to provide their own verification data alongside studies using a model. The test pipeline is included in the base image for each model and can be started through the “mhub.test” entry point.

### CI/CD

Continuous integration and continuous deployment (CI/CD) is implemented via Github Automations ^38^ and ensures that every new model or update goes through automated format validation and metadata validation. We have set up an automated deployment pipeline that creates a model from a Pull-Request (PR), runs through the test pipeline and updates the pull requests with the test reports.

### Model Repository

An overview of all 30 models integrated into the model repository as of today is provided in Table 1. For each model, we report key characteristics of user-model interaction, including (i) input data format, (ii) output format, (iii) application interface used for model inference, and (iv) availability and completeness of user documentation. We documented the status for each characteristic of the original model repository (Table 1, A) and of the MHub.ai implementation of the same model (Table 1, B). We defined three levels of standardization and assigned a point system (standardized / accessible = 2, partially standardized / accessible = 1, not standardized / accessible = 0) to allow a direct comparison for each characteristic. An input is defined as standardized (not standardized respectively) when it supports processing DICOM files directly. For models that process DICOM files with limitations (e.g., require manual separation of DICOM images first) were assigned a 1 point (partial standardization). For segmentation models, the output format is defined as standardized if the model provides a DICOMSEG file and as not standardized for all other formats. For feature extraction and prediction models, the output is defined as standardized if the model provides the prediction in a structured, machine readable format (e.g., CSV, JSON, YAML file) including a reference to the input image’s unique series instance identifier, partial if the format is structured but the identifier is missing and not standardized if the output is provided in a raw format (e.g., image file). The CLI is considered standardized when inference for multiple models can be executed using the same command, with consistent specification of input data, output data (if applicable), additional configuration parameters, and the model to be executed. A partial score is assigned when the CLI is consistent in some, but not all, aspects across multiple models. A not standardized score is assigned to custom CLI implementations where the execution command is specific to an individual model. The documentation (user guide) for a model is considered accessible if it describes the model and its usage, including a description or examples of the CLI command required to run inference. A partially accessible score is assigned when the documentation provides only minimal examples or limited explanation, requiring code inspection to understand the inference process. Documentation is considered not accessible when no instructions for running model inference are provided. The scores are highlighted in the table using colors (green = standardized/accessible, yellow = partially standardized/accessible, red = not standardized/accessible). Accumulating the points shows an initial standardization of 36% (86/240 points) and 99% (237/240 points) for the MHub.ai deployment of the same models, marking an improvement of 275% (151 points).

### Datasets and Evaluation

For the lung segmentation demonstration, 422 chest CT scans from patients diagnosed with non-small cell lung cancer (NSCLC) in the NSCLC-Radiomics dataset were downloaded as DICOM files from the National Cancer Institute Imaging Data Commons ^55^ using the Python idc-index package. Expert annotations of the left and right lungs were available for 303 patients. Three MHub.ai lung segmentation models, TotalSegmentator v1 ^28^, LungMask ^31^, and LungLobes ^42^ were executed using the default command-line interface without model-specific adjustments. Where necessary, lobe-level outputs were aggregated into composite left and right lung masks to match the expert annotation format. Dice similarity coefficients were calculated between model outputs and expert segmentations. Paired t-tests were conducted to compare model accuracy between the left and right lungs. LungLobes showed a significant difference (estimate = −0.0115, p < .001), with lower accuracy in the left lung. In contrast, LungMask (estimate = −0.000435, p = .828) and TotalSegmentator (estimate = −0.00123, p = .539) did not show significant differences between the lungs. Model performance was then analyzed based on patient age, after removing 14 patients without age information. Spearman’s rank-order correlations were conducted to examine the relationship between patient age and model accuracy for each segmentation model (n = 590). There was no significant association between age and Dice score for LungLobes (ρ = .013, p = .756). In contrast, small but statistically significant negative correlations were observed for LungMask (ρ = −.132, p = .001) and TotalSegmentator (ρ = −.116, p = .005), indicating that Dice scores decreased slightly for older patients.

### Public Interactive Dashboards

To promote transparency, demonstrate the benefits of combining public data with public AI models, and highlight the value of representing segmentations in DICOMSEG format, we developed an interactive dashboard for exploring the dataset. Users can filter cases by various clinical, acquisition, and AI-derived attributes and visualize individual CT images along with lung segmentations from the expert and the three AI models.

To build the dashboard, we used off-the-shelf components available in Google Cloud. The DICOMSEG files included in the dataset were first uploaded to a Google Cloud Storage bucket, then ingested into a Google Healthcare API (GHC) ^56^ DICOM Store. We used GHC capabilities to export DICOM metadata from the GHC DICOM Store into a Google BigQuery ^57^ table, enabling queries of any DICOM attributes via a Standard Query Language (SQL) interface. We relied on the public BigQuery tables maintained by IDC to join segmentation metadata with the DICOM metadata of the NSCLC Radiomics CT images already available in IDC ^55^. For lightweight data visualization, we deployed an instance of OHIF Viewer ^19^, configured to fetch CT series from the IDC-maintained public DICOM store and merge them with the DICOMSEG lung segmentation series through a lightweight proxy without authentication. Both are retrieved via the standard DICOMweb interface. All these components enabled an interactive dashboard implemented using Google Looker Studio ^58^, as shown in Figure 4e, which is publicly available at https://lookerstudio.google.com/reporting/0c405365-31f5-4714-8b2a-03631e7a0686.

## Figures and Tables

**Table 1:** Comparison of AI models in their original and MHub.ai deployment format.

Model	Type	A | Original				B | MHub.ai			
		Input Data	Output Format	Interface	User Guide	Input Data	Output Format	Interface	User Guide
**TotalSegmentator** ^28^	S	NIFTI	NIFTI	Custom CLI	Yes	DICOM	DICOMSEG	Workflow	Yes
**Platipy** ^39^	S	NIFTI	NIFTI	Custom CLI	Yes	DICOM	DICOMSEG	Workflow	Yes
**BAMF MR Prostate Seg** ^40^	S	NIFTI / DICOM	NIFTI / DICOMSEG	Docker Mount	Yes	DICOM	DICOMSEG	Workflow	Yes
**BAMF CT Kidney Seg** ^40^	S	DICOM	DICOMSEG	Workflow	Yes	DICOM	DICOMSEG	Workflow	Yes
**CT Liver Seg** ^23^	S	NIFTI	NIFTI	nn-UNet CLI	Yes	DICOM	DICOMSEG	Workflow	Yes
**LungMask** ^31^	S	NIFTI / DICOM	NIFTI	Custom CLI	Yes	DICOM	DICOMSEG	Workflow	Yes
**CaSuSt** ^41^	S	NIFTI / DICOM	NIFTI	Custom CLI	Yes	DICOM	DICOMSEG	Workflow	Yes
**Pulmonary Lobes Seg** ^42^	S	MHA / DICOM	MHA	Custom CLI	No	DICOM	DICOMSEG	Workflow	Yes
**Pancreas Seg** ^23^	S	NIFTI	NIFTI	nn-UNet CLI	Yes	DICOM	DICOMSEG	Workflow	Yes
**Tissue-Background segmentation in histopathological whole-slide images** ^43^	S	TIFF	MHA	Custom CLI + Config File	No	DICOM	TIFF	Workflow	Yes
**BAMF CT Liver Seg** ^40^	S	NIFTI / DICOM	NIFTI / DICOMSEG	Docker Mount	Yes	DICOM	DICOMSEG	Workflow	Yes
**Second place in AutoPET challenge: False Positive Reduction Network** (Peng et al., 2023)	S	MHA	MHA	Docker Mount	No	DICOM	DICOMSEG	Workflow	Yes
**Whole Prostate Seg** ^23^	S	NIFTI	NIFTI	nn-UNet CLI	Yes	DICOM	DICOMSEG	Workflow	Yes
**Prostate Transitional and Peripheral Zone Seg (nnU-Net)** ^23^	S	NIFTI	NIFTI	nn-UNet CLI	Yes	DICOM	DICOMSEG	Workflow	Yes
**Foundation Model for Cancer Imaging Biomarkers** ^44^	F	NIFTI	JSON	Custom CLI	Yes	DICOM	JSON	Workflow	Yes
**PI-CAI challenge baseline** (Bosma et al., 2023)	P	MHA	MHA	Docker Mount	No	DICOM	JSON	Workflow	Yes
**Thoracic OAR (nnU-Net)** ^23^	S	NIFTI	NIFTI	nn-UNet CLI	Yes	DICOM	DICOMSEG	Workflow	Yes
**Chest Radiograph Nodule Locator (Node21 Baseline)** ^45^	P	MHA	JSON	Custom CLI + Docker Mount	Yes	DICOM	JSON	Workflow	Yes
**CT Lung cancer risk prediction** ^46^	P	MHA	JSON	Docker Mount	No	DICOM	JSON	Workflow	Yes
**Pancreatic Ductal Adenocarcinoma Detection in CT** ^47^	S+P	MHA	MHA + JSON	Docker Mount	No	DICOM	DICOMSEG + JSON	Workflow	Yes
**PyRadiomics** ^48^	F	NIFTI	JSON / CSV	Custom CLI + Config File	Yes	DICOM	JSON	Workflow	Yes
**Spine Seg (SPIDER Baseline)** ^49^	S	MHA	MHA	Custom Metadata File	Yes	DICOM	DICOMSEG	Workflow	Yes
**BAMF MR Liver Seg** ^40^	S	NIFTI / DICOM	NIFTI / DICOMSEG	Docker Mount	No	DICOM	DICOMSEG	Workflow	Yes
**Prostate Transitional and Peripheral Zone Seg (Prostate158)** ^50^	S	NIFTI	NIFTI	Custom Monai Config	Yes	DICOM	DICOMSEG	Workflow	Yes
**TIGER challenge winner: Team VUNO** ^51^	P	TIF	JSON	Docker Mount	No	DICOM	JSON	Workflow	Yes
**STOIC2021 baseline** ^52^	P	MHA	JSON	Docker Mount	Yes	DICOM	JSON	Workflow	Yes
**BAMF PET CT Breast** ^40^	S	NIFTI / DICOM	DICOMSEG	Docker Mount	Yes	DICOM	DICOMSEG	Workflow	Yes
**SMIT Self-supervised Lung GTV Segmentation** ^53^	S	NIFTI	NIFTI	Custom CLI	No	DICOM	DICOMSEG	Workflow	Yes
**FDG PET/CT Lung and Lung Tumor Annotation** ^40^	S	NIFTI / DICOM	DICOMSEG	Docker Mount	Yes	DICOM	DICOMSEG	Workflow	Yes
**MRSegmentator** ^54^	S	NIFTI / MHA / NRRD / DICOM	DICOMSEG	Custom CLI	Yes	DICOM	DICOMSEG	Workflow	Yes

Green = standardized / accessible, red = not standardized / not accessible, yellow = partially standardized / partially accessible. The model type is S = segmentation, P = prediction, F = feature extraction.

## Software Availability

- Code, workflows, and containers available at https://github.com/MHubAI.
- Web interface, model metadata, and documentation available at https://mhub.ai/.
- 3D Slicer plugin (MHubRunner) available via the Slicer extension manager and at https://github.com/MHubAI/SlicerMHubRunner.
- Interactive dashboards with lung segmentations, Dice scores, and clinical data available under https://lookerstudio.google.com/reporting/0c405365-31f5-4714-8b2a-03631e7a0686.
